# Ambulatory assessment of walking balance after stroke using instrumented shoes

**DOI:** 10.1186/s12984-016-0146-5

**Published:** 2016-05-19

**Authors:** Fokke B. van Meulen, Dirk Weenk, Jaap H. Buurke, Bert-Jan F. van Beijnum, Peter H. Veltink

**Affiliations:** Biomedical Signals and Systems, MIRA - Institute for Biomedical Technology and Technical Medicine, University of Twente, PO Box 217, Enschede, 7500 AE The Netherlands; Centre for Telematics and Information Technology, University of Twente, PO Box 217, Enschede, 7500 AE The Netherlands; Roessingh Research and Development, Roessingh Rehabilitation Hospital, Roessinghsbleekweg 33b, Enschede, 7522 AH The Netherlands

**Keywords:** Ambulatory assessment, Stroke, Walking balance, Kinematics, Kinetics, Berg balance scale

## Abstract

**Background:**

For optimal guidance of walking rehabilitation therapy of stroke patients in an in-home setting, a small and easy to use wearable system is needed. In this paper we present a new shoe-integrated system that quantifies walking balance during activities of daily living and is not restricted to a lab environment. Quantitative parameters were related to clinically assessed level of balance in order to assess the additional information they provide.

**Methods:**

Data of 13 participants who suffered a stroke were recorded while walking 10 meter trials and wearing special instrumented shoes. The data from 3D force and torque sensors, 3D inertial sensors and ultrasound transducers were fused to estimate 3D (relative) position, velocity, orientation and ground reaction force of each foot. From these estimates, center of mass and base of support were derived together with a dynamic stability margin, which is the (velocity) extrapolated center of mass with respect to the front-line of the base of support in walking direction. Additionally, for each participant step lengths and stance times for both sides as well as asymmetries of these parameters were derived.

**Results:**

Using the proposed shoe-integrated system, a complete reconstruction of the kinematics and kinetics of both feet during walking can be made. Dynamic stability margin and step length symmetry were not significantly correlated with Berg Balance Scale (BBS) score, but participants with a BBS score below 45 showed a small-positive dynamic stability margin and more asymmetrical step lengths. More affected participants, having a lower BBS score, have a lower walking speed, make smaller steps, longer stance times and have more asymmetrical stance times.

**Conclusions:**

The proposed shoe-integrated system and data analysis methods can be used to quantify daily-life walking performance and walking balance, in an ambulatory setting without the use of a lab restricted system. The presented system provides additional insight about the balance mechanism, via parameters describing walking patterns of an individual subject. This information can be used for patient specific and objective evaluation of walking balance and a better guidance of therapies during the rehabilitation.

**Trial registration:**

The study protocol is a subset of a larger protocol and registered in the Netherlands Trial Registry, number NTR3636.

## Background

Impaired walking balance commonly follows a stroke, which reduces the patient’s ability to walk and hence their independence in daily life [1]. Clinical assessment methods of walking balance have been developed to grade a patient’s ability to walk (independently) after stroke [2]. Frequently used assessment scales result in ordinal values, which do not objectively and quantitatively describe balance during walking. These assessment scales only quantify walking balance during prescribed conditions, while knowledge about underlying balance mechanisms is often lacking [3]. This knowledge is mandatory for a better guidance during the rehabilitation of walking and subsequent assessment of walking balance performance during daily life. However, existing systems for quantitative assessment of balance during walking are lab restricted or can only be used for a limited number of steps. For a better guidance during the rehabilitation of walking in a daily life setting, a wearable sensing system that qualitatively evaluates walking balance is needed [4]. This system should quantitatively estimate parameters to describe the movements of the patients’ feet and body center of mass (CoM) during walking in a daily life setting [5, 6]. Preferably, such a system has small-embedded sensors which do not interfere with daily life body movements and behavior [7].

During walking, the CoM is moving within the area between both feet (i.e., base of support, BoS). To evaluate a persons’ stability during walking the extrapolated center of mass (XCoM) can be calculated, which is the position of the CoM extrapolated using the velocity of the CoM. A person will be dynamically stable when the vertical projection of the XCoM on the ground is within the BoS [8–10]. Moments of dynamic instability need to be followed by another step to prevent a fall [8, 10]. These moments of instability normally occur during walking and are necessary for forward progression. A decrease of the distance between BoS and the vertical projection of the XCoM is related to a lower walking speed or a more affected walking pattern [8]. Objective evaluation of walking balance parameters during daily life contributes to insight in underlying mechanisms of balance during community ambulation.

To continuously assess the dynamic stability of a person, information on the position of the XCoM relative to the BoS is necessary. For a continuous evaluation of the BoS, information on movement of both feet relative to each other is required. A feasible method for movement assessment in a daily life setting is the use of inertial measurement units (IMUs). This allows easy assessment of foot movements in a daily life setting without the use of an external physical reference system [11]. Previous studies reported on the use of IMUs for the estimation of qualitative and quantitative parameters of walking and balance performance, such as cadence, stride length and velocity [5, 12, 13]. However, using only IMUs it is not possible to accurately evaluate parameters which depend on the relative position of both feet, such as step length, step width and size of the BoS. By their physical working principle, IMUs do not provide information about relative positions between sensors, only about changes of position of the same sensor. This problem can be solved by fusing data of IMUs and feet distance estimates of an ultrasound sensors system [5].

For a continuous evaluation of CoM position as well as the XCoM, ground reaction forces (GRF) beneath both feet should be known [14] in addition to relative positions of both feet. For the estimation of the GRF beneath both feet, traditionally, multiple force plates or sensorised walkways are used in a lab situation [8, 15, 16]. These systems mostly cause restriction in walking or are only able to measure one or two steps. For the evaluation of forces underneath both feet during daily life activities, shoes instrumented with force or pressure sensors have been investigated in several studies [17–21]. These shoe integrated sensor systems allow ambulatory estimation of ground reaction forces, making it suitable for monitoring multiple steps and walking with changes in walking direction. However, there is no system available that allows the assessment of dynamic stability in a daily life setting and over multiple steps. Such a system would require an ambulatory estimation of foot orientations, relative foot positions and ground reaction forces simultaneously.

The objective of this study is to develop a method to assess balance dynamics during gait in stroke patients in an ambulatory setting and to relate our balance metrics to standardized clinical stability parameters in order to assess the additional information they provide. For this purpose shoes, integrated with force and inertial sensing and ultrasound transducers were combined into a wearable gait measurement system. Quantitative parameters such as the dynamic stability margin, as well as additional temporal, kinematic and kinetic gait parameters will be estimated using the system. Finally, these parameters were related to a frequently used clinically assessment scale of balance, the Berg balance scale (BBS), to evaluate the predictability of the different parameters by clinically-assessed levels of balance.

## Methods

### System setup

**Fig. 1:**
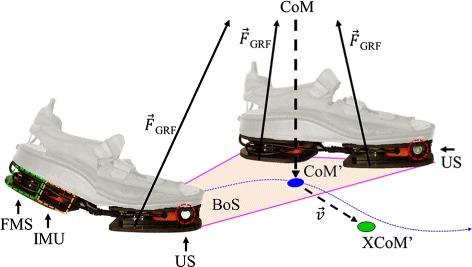
Measurement setup. In this study Xsens ForceShoes™ were used, sandals with underneath one inertial measurement unit (IMU, *dashed orange square*) and one force/moment (FMS, *dashed green square*) sensor per heel or forefoot segment. Near the IMU in the forefeet an ultrasound transducer (US, *dashed red circle*) was mounted in each shoe. Kinematic and kinetic data were used to estimate the position of the center of mass (CoM) relative to the position of both feet, the projection of the center of mass on the ground (CoM’-*blue circle*) within the base of support (BoS) and the extrapolated CoM (XCoM’ - *green circle*)

The ambulatory measurement system used in this study consists of Xsens ForceShoes™ (Xsens Technologies B.V., Enschede, The Netherlands) additionally equipped with ultrasound sensors. All sensors are integrated into an extra sole underneath a pair of sandals. Per foot, each forefoot and heel segment contain one inertial measurement unit (IMU) and one 3D force/moment sensor, see Fig. 1. Only data of the IMUs in the forefoot segments were used. Data of the two IMUs and four force/moment sensors were collected with a sample frequency of 50 Hz. The distance between the feet was estimated using two ultrasound transducers that were mounted near the IMUs in the forefoot segment (Fig. 1). The distance between both shoes was estimated by measuring the time of flight of a 40 kHz ultrasound pulse, that was sent from one shoe to the other. Accurate distance measurements were done approximately 13 times a second [22].

### Participants

For this study seventeen stroke patients from Roessingh rehabilitation hospital, located in Enschede, the Netherlands, were recruited. Recruited participants were between 35 and 75 years of age and had a hemiparesis as a result of a single unilateral ischemic or hemorrhagic stroke, diagnosed at least six months earlier. Exclusion criteria were inability to perform given instructions, inability to understand questionnaires, a medical history with more than one stroke or another medical history which might negatively influence the participant’s walking pattern. The study protocol is a subset of a larger protocol approved by the local medical ethics committee (METC Twente, the Netherlands, P12-27) [11]. The whole study is registered in the Netherlands Trial Registry, NTR3636. All participants signed written informed consent before participating.

Two participants with severely affected lower extremity function were not able to complete the task without assistance due to unstable walking patterns. The corresponding measurements were excluded from the analysis. Data of two other participants were not fully recorded because of a broken cable during the session or sensors that were not properly functioning. Remaining were 13 participants (8 male and 5 female) with an average age of 64.1(SD ± 8.7) years, 2.4 (SD ± 1.8) years post stroke. Participant-specific information is reported in Table 1 and includes gender, age, number of years post stroke, dominant and affected side, weight, height, BBS score and whether or not a walking aid is normally used during activities of daily living. Participants were ranked from low to high BBS score.

**Table 1:** General participant characteristics

ID^a^	Gender	Age^b^	Post	Dominant	Affected	Weight^c^	Height^d^	BBS^e^	Walking
			stroke^b^	side	side				aid^f^
1	M	54	2.9	R	L	109	1.74	35	St, AFO
2	M	69	4.0	R	L	96	1.90	42	–
3	F	67	3.3	R	L	80	1.62	43	St
4	M	75	1.6	R	L	88	1.72	45	St
5	F	55	1.4	R	L	87	1.68	49	–
6	M	70	7.4	R	L	94	1.74	52	–
7	M	65	1.3	R	L	92	1.86	52	OS
8	M	70	1.2	L	L	99	1.81	52	–
9	M	47	1.8	R	L	88	1.73	54	–
10	M	73	2.4	R	R	82	1.78	54	–
11	F	60	0.7	R	L	74	1.65	55	–
12	F	71	1.4	R	R	67	1.53	56	–
13	F	56	1.6	R	L	85	1.74	56	OS

^a^Participant identification number (participants are ranked from a low to high BBS score)

^b^in years

^c^in kilograms

^d^in meters

^e^Berg Balance Scale score (0–56 points)

^f^Use of walking aid during activities of daily living: St = Stick, AFO = Ankle foot orthosis, OS = Orthopedic Shoes

### Experimental protocol

Participants performed twice a timed 10 meter walk at a self-selected comfortable pace along a 10 meter path [23], while wearing the instrumented shoes and without the use of any walking aid. To relate results of the new setup with a frequently used clinically assessment scale to assess balance, participants’ balance was evaluated using the Berg balance scale (BBS) [24]. All assessments were performed by the same technical physician who has adequate clinical expertise to perform the assessment.

### Data processing

#### Kinematic data

**Fig. 2:**
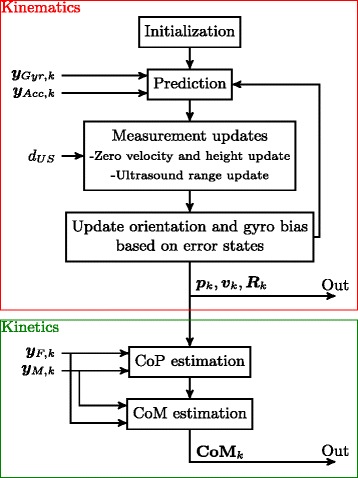
Sensor fusion. The upper part (kinematics) is an extended Kalman filter that fuses the signals from the accelerometer (***y***_*Acc*_) and gyroscope (***y***_*Gyr*_) and applies zero velocity, height and ultrasound range measurement updates (*d*_*US*_). Outputs are 3D position (***p***), velocity (***v***) and orientation (***R***) estimates of the forefoot segments. For kinetic estimation, data from the 3D force/moment sensors (***y***_*F*_ and ***y***_*M*_) are used to estimate 3D CoM. With ***y***_*M*_) are used to estimate 3D CoM. With subscript *k* the samples are indicated. The estimation frequency is 50 Hz and ultrasound range updates are applied at approximately 13 Hz

All data were processed offline and analyzed using MATLAB$^{\circledR }$ (MathWorks Inc., Natick, MA). Three dimensional (3D) positions (***p***), velocities (***v***) and orientations (***R***) were estimated using an extended Kalman filter (upper part of Fig. 2). The filter fuses ultrasound range estimates (*d*_*US*_), essential for estimating relative foot positions, with 3D accelerations (***y***_*Acc*_), 3D angular velocities (***y***_*Gyr*_), with the goal to estimate the state vector:

1 $$\begin{array}{@{}rcl@{}}  \boldsymbol{x} &=& \big(\ \boldsymbol{p}_{r}\ \ \boldsymbol{p}_{l}\ \ \boldsymbol{v}_{r}\ \ \boldsymbol{v}_{l}\ \ \boldsymbol{\theta}_{\epsilon,r}\ \ \boldsymbol{\theta}_{\epsilon,l}\ \ \boldsymbol{b}_{\epsilon,r}\ \ \boldsymbol{b}_{\epsilon,l}\ \big)^{T} \end{array} $$

with position, velocity, orientation error (***θ***_*ε*_) and gyroscope bias error (***b***_*ε*_) of each IMU. The subscripts *r* and *l* indicates respectively the right and left foot. The filter starts with an initialization in which the initial positions and orientations are set based on the accelerometer signal and the initial ultrasound range, assuming the patient is standing with both feet flat on the floor. When a step is made, the 3D position, velocity and orientations of right and left forefoot are predicted using the IMU data. After this prediction, two measurement updates are performed. First, height-, and velocity are updated to be zero when the foot is in contact with the ground, which is detected using the method presented by Skog and others [25]. Second, when an accurate ultrasound range estimate is available, estimated using:

2 $$\begin{array}{@{}rcl@{}}  d_{US} &=& v_{s}\ \cdot\ t_{ToF} \end{array} $$

based on the speed of sound (*v*_*s*_) and the time of flight (*t*_*ToF*_) of an ultrasound pulse between both transducers, the position of the (last) moving foot is updated according to the estimated range. This estimated range is equal to the distance between both feet:

3 $$\begin{array}{@{}rcl@{}}  ||\ \boldsymbol{p}_{r}\ -\ \boldsymbol{p}_{l}\ || = d_{US} \end{array} $$

Subsequently, the orientation and gyroscope bias are updated based on the error states. More details can be found in [5].

These algorithms were validated in healthy subjects using an optical reference system. Mean absolute differences in estimated step lengths and step widths were below 2 cm [5] and mean absolute differences in estimated feet distances were below 1 cm [22].

#### Kinetic data

The trajectories of the center of pressure per foot (*CoP*_*r*_ or *CoP*_*l*_) in the global frame were estimated using measured forces (***y***_*F*_) and moments (***y***_*M*_) of the two force/moment sensors of one foot, using:

4 $$\begin{array}{@{}rcl@{}}  {CoP}_{i} &=& \left(\begin{array}{c} -\frac{M_{y,i}}{F_{z,i}} \\ \frac{M_{x,i}}{F_{z,i}} \\ 0 \end{array}\right) \end{array} $$

in which subscript *i* indicates the right or the left foot, *F*_*z*,*i*_ is the vertical component of the GRF and *M*_*x*,*i*_ and *M*_*y*,*i*_ are the horizontal components of the moments [14]. After combining (relative) foot positions (***p***) and estimated CoP trajectories of each foot, the total CoP was estimated by weighting the CoP trajectories of the right (*CoP*_*r*_) and left (*CoP*_*l*_) foot by the relative magnitude of the GRF of the right (***F***_*r*_) or the left (***F***_*l*_) foot:

5 $$\begin{array}{@{}rcl@{}}  CoP &=& \frac{||\boldsymbol{F}_{r}||}{||\boldsymbol{F}_{l} + \boldsymbol{F}_{r}||}{CoP}_{r} + \frac{||\boldsymbol{F}_{l}||}{||\boldsymbol{F}_{l} + \boldsymbol{F}_{r}||}{CoP}_{l} \end{array} $$

Knowing the relative foot positions and the position of the total CoP, the position of the CoM was obtained using the method of Schepers and others [14]. In this method the CoM position estimation is a summation of the low-pass filtered component of the total CoP movement and the high pass filtered component of the double integrated CoM acceleration (lower part of Fig. 2).

Schepers and others evaluated their method in which they assume a known relative distance between both feet (|| ***p***_*r*_−***p***_*l*_ ||), by comparing their method and an optical reference system in seven stroke patients. They found small positional differences between methods, rms values were equal or below 2 cm (± 0.7 cm) in all directions [14].

### Data analysis

#### Parameter selection

Hemiparetic stroke patients use different walking strategies to stay comfortable and in balance. To be able to compensate for their reduced coordination of their affected side, they often reduce their walking speed, make shorter steps, a longer stance time on their non-affected side and lean more towards their non-affected side [26–29]. This results in a more asymmetrical walking pattern of the more affected patients. Using the complete kinematic and kinetic reconstruction during walking, temporal, kinematic and kinetic parameters can be calculated to quantify these typical walking patterns.

**Fig. 3:**
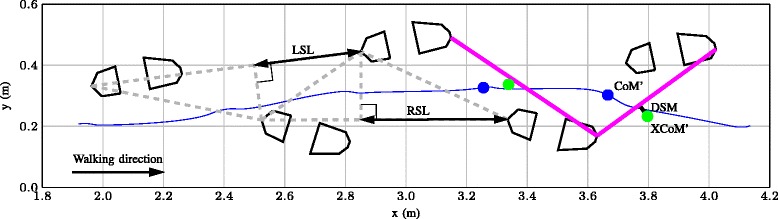
Top-down view of foot positions. Top-down view of foot positions from four steps of participant number 3. For both left and right foot, the step lengths (LSL and RSL respectively) are calculated using triangles obtained from the positions during stance and indicated in the left part of the figure. The CoM’ and its trajectory (*blue*) and the XCoM’ (*green*) together with the front line of the BoS, just before heel-off (*pink*) are shown on the right. The shortest distance from the XCoM’ to the front line of the BoS (in walking direction, to the right in this figure) is calculated (DSM) and indicated in the figure with a *black line*

First, walking speed was calculated as the average velocity of both feet during walking, estimated with the extended Kalman filter. As a reference of the current protocol, walking speed was estimated by measuring the duration of 10 meter walking using a stopwatch, which includes gait initiation. Next, stance times were calculated for the affected and non-affected side. Stance times were defined as the period between first contact of the foot (heel or forefoot) with the ground until end of contact of the foot. Contact of the forefoot and heel segments with the ground were evaluated per segment at any time, by thresholding the magnitude of the 3D force at 20 Newton. From the estimated 3D positions of the left and right foot, step lengths (LSL and RSL respectively) were calculated using the method as described by Huxham and others [30] (see Fig. 3).

Asymmetries in stance times and step lengths between the non-affected and the affected side were calculated using:

6 $$\begin{array}{@{}rcl@{}}  SI = \frac{p_{A} - p_{N}}{p_{N}} \end{array} $$

with *SI* the symmetry index value, *p*_*A*_ the parameter value for the affected side and *p*_*N*_ the parameter value for the non-affected side. Larger positive and negative values indicate a greater asymmetry towards the affected and non-affected side respectively. *SI* values equal to zero, indicate a perfect symmetry.

Furthermore, position and velocity of the CoM relative to the BoS were evaluated. The participants’ BoS was defined by the area between all foot segments which were in contact with the ground. Knowing the position of the CoM (relative to the BoS) and the velocity of the CoM, the XCoM’ was calculated as [10]:

7 $$\begin{array}{@{}rcl@{}}  \text{XCoM'} = \text{CoM'} + \frac{v_{\text{CoM}}}{\omega_{0}} \end{array} $$

with CoM’ the position of the vertical projection of the CoM on the ground, *v*_CoM_ the velocity of the CoM in the transversal plane and $\omega _{0} = \sqrt {g/l_{0}}$, in which *g*= 9.81 m/s ^2^ (earth gravitational acceleration) and *l*_0_ the greater trochanter height, as we estimated from a proportion of the total body height [31]. Knowing XCoM’ relative to BoS, a dynamic stability margin (DSM) was calculated as the shortest distance from the XCoM’ to the front line (in the walking direction) of the BoS. When one foot is in swing phase, i.e., the BoS is restricted to the size of only the other foot, no estimation of DSM was made.

We define negative DSM values as dynamically stable, the XCoM’ is within the BoS and positive DSM values as dynamically unstable, the XCoM is outside the BoS. Figure 3 shows a top down view of four consecutive steps of a walking trial of participant #3. In this figure XCoM’ and the front-line of the BoS just before heel-off are indicated, including the shortest distance between them.

#### Statistical analysis

To exclude gait initiation and termination steps, from each of the two walking trials per participant, the first two and last two steps were removed. For both walking trials of each participant the mean of all parameters was calculated per side. The mean DSM was not calculated per side. To be able to compare different participants, parameters were normalized to body size as described by Hof and others [32]. Velocity values were normalized to $v_{0} = \sqrt {g l_{0}}$, stance times to $t_{0} = \sqrt {l_{0}/g}$ and step length and DSM values to *l*_0_.

Linear regression analysis using Pearson’s correlation coefficients (r) was performed to relate the different parameters with the clinically-assessed levels of balance. The temporal and kinematic parameters, were taken as dependent variables, and the BBS score (assessed using the instrumented shoes) as independent value. When investigating correlation between BBS with symmetry indices, the absolute value was used, to neglect to which side the asymmetry occurs. Statistical significance was determined as a *p*-value of less than 0.05. The explained variance (*R*^2^) was calculated and assumed to be low when this value is less than 0.5, i.e., less than 50 % of the variance can be explained by the linear regression model.

## Results

For all participants the normalized walking speed in walking direction (*v*_*n*_) was estimated. Table 2 shows the mean velocity during the selected steps for each participant, as estimated by the extended Kalman filter (*v*). As a reference, the velocity estimated from the stopwatch (*v*_*ref*_) of the complete timed 10 meter walk is also listed. More-affected participants with a lower BBS score show a significantly lower walking speed (*r* = 0.71, *p* < 0.01). All correlation values (*r*) of the different parameters and BBS, their significance levels and the explained variance (*R*^2^) are presented in Table 3.

**Table 2:** Velocity for each participant

ID^a^	BBS^b^	*v*_*ref*_	*v* ^⋆^	*v*_*n*_^◇^	*S* *I*_Stance_	*S* *I*_Step_
1	35	0.43	0.49	0.15	–0.16	0.16
2	42	0.62	0.68	0.21	–0.19	0.08
3	43	0.54	0.58	0.16	–0.13	–0.33
4	45	0.60	0.63	0.19	–0.19	0.02
5	49	0.74	0.87	0.25	–0.05	–0.06
6	52	0.76	0.86	0.26	–0.20	0.04
7	52	0.94	1.03	0.32	0.01	0.07
8	52	0.91	1.13	0.35	–0.04	0.03
9	54	0.95	0.99	0.30	–0.13	–0.08
10	54	0.96	1.11	0.34	–0.03	–0.05
11	55	1.28	1.45	0.41	0.00	0.01
12	56	0.63	0.73	0.20	–0.08	0.12
13	56	0.83	0.96	0.28	–0.08	–0.02

^a^Participant identification number

^b^Berg Balance Scale score (0-56 points). *v*_*ref*_: velocity (m/s) calculated from the time to pass 10 meters, measured using a stopwatch. *v* ^⋆^: velocity during selected steps (m/s) estimated by the extended Kalman filter. *v*_*n*_^◇^: velocity *v* ^⋆^normalized to $v_{0} = \sqrt {g l_{0}}$. *S* *I*_Stance_ symmetry index value for stance times. *S* *I*_Step_ symmetry index value for step lengths

**Table 3:** Relation quantifying parameters and BBS

Parameter (related to BBS^1^)	*r*	*R* ^2^	*p*
Velocity	0.71	0.50	<0.01
Stance time affected side	–0.69	0.48	<0.01
Stance time non-affected side	–0.80	0.64	<0.01
Symmetry Index stance time	–0.58	0.34	<0.05
Step length affected side	0.77	0.59	<0.01
Step length non-affected side	0.74	0.55	<0.01
Symmetry Index step length	–0.51	0.26	0.074
Dynamic stability margin	0.41	0.17	0.168

^a^Berg Balance Scale score. *r* is Pearson’s correlation coefficient and *R* ^2^ the explained variance. *p* values <0.05 indicates significant correlations

**Fig. 4:**
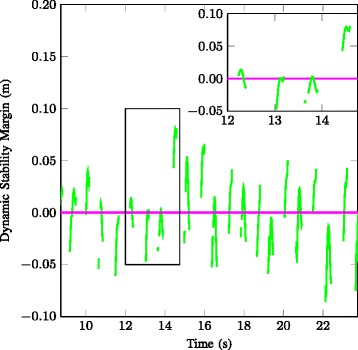
Example of the dynamic stability margin over time. The the DSM evaluated during the double stance phases for one walking trial of participant number 3. Negative values indicate that the XCoM’ is inside the BoS. Mean DSM for this trial was 0.00 m. The inset is a magnification (indicated by the box) and corresponds to the time window (12.00-14.75 s) of which the steps are shown in Fig. 3

Figure 4 shows the dynamic stability margin of participant #3, during the selected steps of a single walking trial, over time. If one foot is in swing phase no estimation of DSM is made, which is represented as a gap in Fig. 4. The four steps that are shown in Fig. 3 are indicated by the rectangular box and a zoom of these steps is shown in the inset of the figure. The mean DSM of this trial is 0.00 m.

**Fig. 5:**
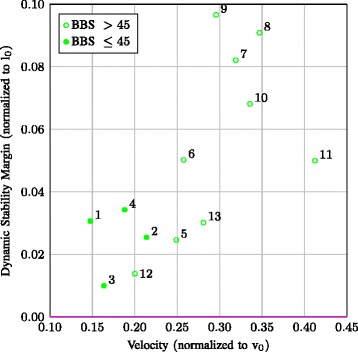
Mean DSM versus velocity. Mean DSM (normalized to *l*_0_) versus velocity (normalized to *v*_0_) estimated by the filter (*v*_*n*_ in Table 2), for all 13 participants (indicated with the numbers). Numbers indicate participant identification number, which are ranked from a low to high BBS score. Filled data markers are of those participants with a BBS score below or equal to 45

The normalized mean DSM values were estimated for both walking trials of each participant and related to participant’s average normalized walking speed (*v*_*n*_), as shown in Fig. 5. The average DSM is positive, i.e., XCoM’ is outside the BoS. Especially participants with lower BBS scores show a lower walking speed and small positive mean DSM values. No significant correlation between BBS and DSM was found (*r*=0.41, *p*=0.167).

**Fig. 6:**
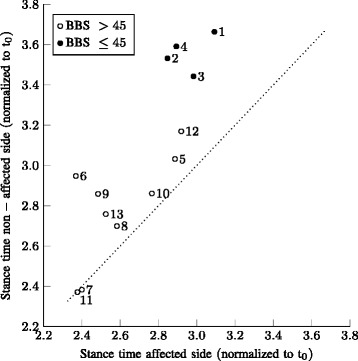
Stance time of the affected side versus the non affected side. Mean stance time for affected versus non-affected side (normalized to $t_{0} = \sqrt {l_{0}/g}$). Numbers indicate participant identification number, which are ranked from a low to high BBS score. Filled data markers are of those participants with a BBS score below or equal to 45. Both trials of a patient are averaged. The line x = y is plotted to indicate a symmetric walking pattern

Results of the mean normalized stance times for the affected side versus the non-affected side are shown in Fig. 6. Overall, participants show a longer stance time on their non-affected leg and participants with lower BBS scores (below 45) show longer stance times for both sides. Furthermore, more asymmetry in stance times is visible for the participants with a lower BBS score (see also Table 2). Although participants 6 and 9 (having higher BBS scores) show large asymmetries in stance time as well, the asymmetry in stance times significantly correlates with the BBS score (*r*=−0.58, *p*<0.05).

**Fig. 7:**
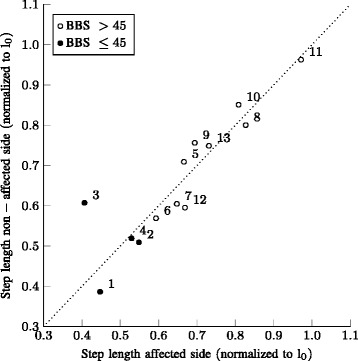
Step length of the affected side versus the non affected side. Mean step length for affected versus non-affected side (normalized to leg length *l*_0_). Numbers indicate participant identification number, which are ranked from a low to high BBS score. Filled data markers are of those participants with a BBS score below or equal to 45. Both trials of a patient are averaged. The line x = y is plotted to indicate a symmetric walking pattern

The mean normalized step lengths for the affected side versus the non-affected side are shown in Fig. 7. The step lengths are relatively symmetric, except for the two trials of participant number 3 (±0.4 versus ±0.6 normalized step length for the affected and non-affected side respectively). Overall the step lengths are smaller for participants with a smaller BBS score. The asymmetry in step length (see Table 2) is not significantly correlated with the BBS score (*r*=−0.51, *p*=0.074).

## Discussion

The objective of this study was to develop a method to quantitatively assess balance dynamics during gait in stroke patients in an ambulatory setting. Our balance metrics were related to standardized clinical stability parameters (i.e., BBS scores) in order to assess the additional information they provide. By combining Xsens ForceShoes™ and ultrasound modules, we were able to completely reconstruct kinetics and kinematics of both feet as well as the position of the CoM relative to both feet during walking, without the use of a lab restricted setup. All underlying physical parameters of the presented system have been validated against a gold standard [5, 14, 22].

Although no parts of the BBS include assessment of walking, a high correlation was found between walking speed and BBS scores. As in Liston, et al. [27, 33], participants show a higher walking speed with an increase of BBS score. During walking, step lengths and stance times for both the affected side and the non-affected side show correlations with the BBS scores. Participants with a higher BBS score, show an increase of step length on both sides and a decrease of stance times on both sides. A significant negative relation in stance time asymmetry and BBS was found, stance times of both sides are getting more symmetrical with a higher BBS score. However, no significant relation between step length symmetry and BBS was found. These results are contradictory to earlier findings of Lewek and others [16]. Compared to our study, they describe slightly different correlation values out of which they conclude the presence of a negative (weak-to-moderate) correlation between BBS and step length asymmetry and the absence of a relation between BBS and stance time asymmetry measured using a sensorised walkway. These different outcomes could be related to, for example the difference between average age and number of months post stroke of the groups of participants or the larger sample size compared to our study. The sample size in our study is relatively small and the participants’ BBS scores are limitedly distributed, which is a limitation of our study.

By extrapolating the CoM’ using its velocity, the XCoM’ was estimated. This XCoM’ can be used to examine stability during walking, by evaluating the distance between XCoM’ and the front-line of the BoS (i.e., DSM). Overall, participants with higher BBS scores show larger (as expected, however not significantly larger) average DSM values. More-affected participants – especially the ones with BBS score of 45 or lower, who have a higher risk of falling – show smaller velocities and smaller and almost negative average values for their DSM. In case of a negative mean DSM value during walking, a person is dynamically stable during walking, which means that after each step made, no extra step is needed to prevent a fall (on average). Nevertheless, positive DSM values, e.g., moments of dynamical instability, are necessary for forward progression. Participants with a lower BBS score might decrease their average DSM value as a conservative balance strategy in order to be more stable during walking. Although, this may cause interruptions in walking and possible risk of falling backwards [34]. Furthermore, a smaller walking velocity may be less efficient [35].

By estimating the walking speed, asymmetry in walking and especially the size of patients’ DSM during walking, it may be possible to objectively follow up improvement or deterioration of daily life ambulation. These parameters offer additional information not only on activity level (using the BBS) but also on the level of body function. This information may be of importance during rehabilitation training, because it provides extra information on impairment level (during a functional task). Monitoring these parameters adds insight whether or not changes on ability level are associated with changes on impairment level. Thus providing insight whether improvement is due to restoration of body function or whether these changes are related to compensation and adaptive strategies are used to overcome the problems on impairment level. The ability to control the position of the XCoM’ with respect to the BoS might for instance be a compensatory mechanism for preventing falls during walking [34]. Furthermore, using the presented system it is possible to gather patient specific information. Although most parameters are significantly related to the BBS, when evaluated in a group of stroke patients, the explained variances (*R*^2^, see Table 3) are low. Therefore this patient specific information estimated using the described setup, cannot accurately be predicted by just evaluating the BBS score. The additional information such as average walking speed and DSM value during walking, along with clinically evaluated balance scores, can be used as a guidance for patient specific clinical practice. For instance, if a patient shows high clinically evaluated balance scores but small DSM values, increased walking speed may be advised. Alternatively, a patient who shows large DSM values but low clinical balance scores, might have a higher risk of falling and should be advised to adapt their walking pattern to their balance capacity. This approach should be evaluated in future research to demonstrate the effectiveness of using these parameters for the guidance in rehabilitation practice.

In addition to the average DSM values over multiple double stance phases, the time course of the DSM value (as shown in Fig. 4) may provide additional insight in walking balance and continuity of the walking pattern. In case of a negative DSM value at the beginning of a double stance phase gait can be terminated without an additional step. However, if the minimum value of the DSM during the double stance phase is positive, an additional step is always needed before gait can be terminated.

Future research should focus on the sizes of the used sensors, which were integrated in the instrumented shoes. Although previous research found only limited influence on walking patterns of patients with knee osteoarthritis while wearing these instrumented shoes [21], walking might be more exhausting and chances of a trip are higher by the design of the shoes. Currently the shoes are relatively heavy (±1 kg per shoe) and the sole height is relatively high (±2.5 cm). New technical developments in the use of smaller and lighter force/moment sensors [36] integrated in shoes and the already widely available smaller inertial sensors, may result in instrumented shoes that can be used in daily life [7]. In addition, the number of sensors may be reduced depending on the actual research question. We presented a system using one IMU, two force/moment sensors and an ultrasound transducer per shoe (data of the inertial sensors in the heel part of the shoes was not used), which are all required for the dynamic balance parameters shown in Figs. 4 and 5. Using a reduced set of sensors, several relevant objective parameters can still be determined. For example, for the estimation of stance and swing times (as in Fig. 6), a system with only inertial sensors or only 1D force sensors could be used. For the evaluation of step lengths and step widths (as in Fig. 7), a system with inertial sensors in combination with ultrasound transducers suffices, as was shown in [37]. Although not used in the presented methods, the inertial sensor data of the heel segments can be used to additionally evaluate orientations of heel segments, the rolling of the feet or heel contact times.

Besides the evaluation of straight line walking, it is possible to evaluate other phases of walking, such as gait initiation and termination, standing, transfers, turning and non-repetitive walking patterns. Especially in an ambulatory setting, the stability of a stroke patient during these phases might be of interest, because of the high incidence of falls during these non-stationary walking phases [38]. In addition to stroke patients, the presented system might be of interest in other groups of patients with difficulties in walking (e.g. before and after knee or hip surgery).

## Conclusions

We demonstrated a method to assess walking balance in stroke patients under ambulatory conditions. Using the described setup, objective evaluation of walking is no longer restricted to a lab setting. Quantitative parameters can be used for describing walking patterns of the individual patient. DSM values and the asymmetry in step lengths, are not significantly correlated with participants BBS scores. Walking velocity, step lengths of both feet, stance times of both feet and the asymmetry in stance time are significantly correlated with participants BBS score, although the explained variance of the velocity of walking, stance time on the affected side and asymmetry in stances time is limited to approximately 0.5. The presented system provides important information about the walking balance in addition to parameters describing the walking pattern of an individual subject, which is only partly predictable in the individual person using BBS.

## Abbreviations

BBS: Berg balance scale
BoS: base of support
CoM: center of mass
CoP: center of pressure
DSM: dynamic stability margin
GRF: ground reaction force
IMU: inertial measurement unit
LSL: left step length
RSL: right step length
XCoM: extrapolated center of mass
